# Insights from Eye-Tracking Technology in Infants with or at Risk of a Motor Disability: A Systematic Review

**DOI:** 10.3390/children13081011

**Published:** 2026-07-30

**Authors:** Sabrina Schaly, Annemarie Murphy, Darryl Chiu, Alistair McEwan, Petra Karlsson

**Affiliations:** 1 School of Biomedical Engineering, The University of Sydney, Sydney, NSW 2008, Australiapkarlsson@cerebralpalsy.org.au (P.K.); 2 Cerebral Palsy Alliance, Camperdown, NSW 2050, Australia

**Keywords:** eye-tracking technology, eye gaze, infant development, motor disability, early intervention, pediatric

## Abstract

**Highlights:**

**What are the main findings?**

- Eye-tracking can measure how infants, with or at risk of motor disabilities, interact with the world, including their attention, learning, and early communication skills.
- Infants at risk of a motor disability (e.g., preterm or with brain injury) often show slower visual responses and differences in attention, which may be detected using eye-tracking technology, even when standard clinical tests appear normal.

**What are the implications of the main findings?**

- Eye-tracking could be a useful early detection tool to identify developmental delays, allowing earlier and more targeted support for infants at risk.
- There is a need for more consistent and standardized ways of using eye-tracking technology, so results can be compared and applied more reliably.

**Abstract:**

**Background/Objectives:** Limited evidence exists on the use of eye-tracking technology in infants with disabilities, particularly with motor disabilities. This systematic review examines the eye-tracking metrics, protocols, and feasibility of eye-tracking technology in infants at risk or with a motor disability. **Method:** This systematic review was conducted in accordance with PRISMA guidelines and registered on PROSPERO (CRD42024563282). PubMed, Web of Science, CINAHL, ERIC, Embase, Scopus, MEDLINE, and the Tobii database were systematically searched, with two independent reviewers screening studies, extracting data, and assessing methodological quality using the Oxford Centre for Evidence-Based Medicine Levels of Evidence and Standard Quality Assessment Criteria. **Results:** A total of 15 studies were included with a total sample size of 667 (range 1–123), including 341 boys, 306 girls, and 20 infant genders unreported (M = 10.8 months; SD = 4.8; range 4–24 months). The infants included had brain injury as seen via MRI (4 studies), cerebral palsy (4 studies), or were born very preterm with varying risk factors (7 studies). In these studies, eye-tracking technology provided an objective measure of gaze patterns, gaze duration, fixation frequency, visual assessments, and saccades. Despite Tobii being the most common eye-tracking technology used, protocols varied by study design, duration, stimuli, metrics reported, and settings, making standardization challenging. **Conclusions:** Eye-tracking technology provides objective eye-tracking metrics that may be used to assess infant development. However, given the heterogeneity of studies, future research should follow standardized stimuli, protocols, and include diverse populations to make definitive conclusions and implications.

## 1. Introduction

Infant developmental assessments are essential for monitoring early development and facilitating timely support and intervention when delays emerge [1]. The first 1000 days of life represent a critical period for physical and psychological development, during which early identification of developmental risk can inform interventions that prevent or mitigate motor and cognitive impairment [2]. Standardized assessments such as the Bayley Scales of Infant and Toddler Development, Third Edition (BSID-III) [3], the Developmental Assessment of Young Children, Second Edition (DAYC-2) [4], and the Alberta Infant Motor Scale (AIMS) [5] are commonly used to evaluate cognitive, language, motor, and social-emotional development in infancy.

Assessing infant development remains challenging, particularly in early life due to substantial inter- and intra-individual variability [6]. Additionally, in the absence of infants’ verbal communication, developmental assessments rely heavily on observation, which can be subjective and susceptible to assessor bias and variability [7]. These challenges are exacerbated in infants with motor disabilities, for whom limited mobility may mask cognitive, social, or communicative abilities. Recent evidence indicates that only three of nine commonly used cognitive assessment tools are suitable for infants with motor impairments, and that available measures are under researched and demonstrate poor- to fair-quality psychometric properties [8].

Eye-tracking offers a promising alternative approach to assessing infant cognitive, social, and linguistic development without reliance on motor responses. Eye-gaze patterns and related metrics can provide insight into underlying brain function, attention, memory, social development, and joint attention [9]. Beyond assessment, eye-tracking can function as an access method for communication in children with severe physical disabilities, including cerebral palsy [10], and may improve quality of life and communicative participation across the lifespan [11]. Eye-tracking has also been used to support language learning and neural processing by leveraging gaze as an instructional cue [12,13]. Traditional manual eye-tracking methods, however, are time-intensive, prone to measurement error, and difficult to scale or interpret when applied to complex datasets [14]. These limitations, alongside cognitive load for the assessor, training requirements, and infant temperament, have historically constrained the feasibility of eye-tracking-based assessments in infancy.

Advances in eye-tracking technology address many of these challenges by providing a non-invasive, objective, and precise means of measuring visual attention, social engagement, language processing, and visual preference [13]. Eye-tracking technology enables hands-free interaction by tracking and interpreting a user’s visual focus. A dedicated camera emits infrared light and detects its reflection from the cornea and pupil, allowing the system to estimate the direction of gaze with high precision. The user’s gaze is translated into conventional computer inputs, such as cursor movement, a mouse click is typically achieved through a dwell (maintaining gaze on a target for a predefined duration) [15]. While eye-tracking technology is widely applied in typically developing pediatric populations [16], its feasibility, protocols, and clinical utility in infants under 24 months of age, particularly those with motor disabilities, remain unclear.

Despite growing interest in this technology, there is no comprehensive synthesis of the evidence examining eye-tracking technology in infants with motor delays or disabilities. This systematic review addresses this gap by synthesizing current evidence on the use of eye-tracking technologies in infants aged 0–24 months with or at risk of a motor disability. Specifically, this review aims to address the following research questions in infant populations with or at risk of a motor disability: (1) What is the feasibility of eye-tracking technology? (2) What metrics can be obtained using eye-tracking technology? (3) What eye-tracking technology protocols or procedures have been used? By consolidating the available evidence, this review seeks to identify methodological gaps, inform future research directions, and clarify the potential role of eye-tracking technology in early developmental assessment and intervention.

## 2. Materials and Methods

This systematic review followed PRISMA (Preferred Reporting Items for Systematic Reviews and Meta-Analyses) guidelines. The research question was developed prior to conducting the review using Population-Intervention-Comparison-Outcome (PICO) format. The population (P) comprised infants aged 0–24 months with or at risk of developing a motor disability. The intervention (I) was the use of infrared eye-tracking technology for assessment, monitoring, intervention, or communication purposes. The comparison (C) included typically developing infants, alternative assessment methods, different clinical subgroups, or no comparison group, depending on the study design. The outcomes (O) were broad and included any reported eye-tracking measures (e.g., gaze behaviour, fixation duration, saccades, visual attention), developmental outcomes, and feasibility measures given the limited studies available. Next, the study protocol was registered on PROSPERO (ID = CRD42024563282).

### 2.1. Eligibility Criteria

The study must involve the use of eye-tracking technology in infants with or at risk of a motor disability aged 0–24 months. Eye-tracking technology was defined as any technology that uses infrared sensors to measure eye movements. Common commercial brands include Tobii, SR Research, and Smart Eye.

No publication date or study design filters were applied during database searching to prevent any improper study omissions. Only studies in English were included.

Studies involving both typically developing infants and infants with a motor disability were included only if data from the infants with a motor disability could be disaggregated. Many infants are not diagnosed with a motor disability until five years of age [17], thus infants with a high risk of developing a motor disability were also included. Infants were considered at a high risk of developing a motor disability if they met one of the following criteria [18,19]:

- Bilateral parenchymal or intraventricular hemorrhages (grade IV);
- Bilateral cystic periventricular leukomalacia (grade III);
- Brain maldevelopment or lesions as seen on Magnetic Resonance Imaging;
- Basal ganglia injury;
- Unilateral lesions (grade IV hemorrhage or perinatal arterial ischemic stroke);
- Periventricular leukomalacia (non-cystic);
- Moderate to severe white matter injury;
- Reduced motor functionality as assessed by a standardized motor, movement, or neurological assessment (e.g., Bayley Scales of Infant and Toddler Development (BSID-III), Hammersmith Infant Neurological Examination (HINE), Alberta Infant Motor Scale (AIMS), and Developmental Assessment of Young Children, Second Edition DAYC-2);
- Neonatal convulsions or seizures;
- Neonatal infections like chorioamnionitis, sepsis, toxoplasmosis, rubella (German measles), cytomegalovirus, and herpes;
- Respiratory failure or asphyxia;
- Or very preterm (<30 weeks) and also report one of the following risk factors:
  - Mean infant weight is <2000 g at birth;
  - Low Apgar scores at 5, 10 and 20 min;
  - Multiples;
  - Assisted reproductive technology infertility treatments.

### 2.2. Exclusion Criteria

Studies were excluded if (1) information on infants below 24 months old could not be disaggregated; (2) the study only included typically developing infants or; (3) the study included infants with autism or intellectual disability without any motor disability risk factors listed above. Studies that only used manual eye-tracking rather than eye-tracking technology were also excluded. Non-peer-reviewed articles were excluded.

### 2.3. Information Sources

PubMed, Web of Science, CINAHL, ERIC, Embase, Scopus, and MEDLINE were systematically searched to identify relevant studies on the topic published at any time. The reference list of the included studies was hand-searched for additional studies. A library of all publications using Tobii eye-tracking technology (https://www.tobii.com/learn-and-support/scientific-publications?q=infant, accessed on 20 January 2026) was also hand-searched which returned no eligible studies. A final search of all databases was conducted on 20 January 2026.

### 2.4. Search Strategy

Search strategies were adapted to the indexing terms and search functionality of each database. Search terms were developed around three key concepts: (1) motor disability or neurological risk factors; (2) infant populations; and (3) eye-tracking technology. Synonyms and controlled vocabulary terms were adapted for each database. The complete database-specific search strategies are provided in Supplementary File S1.

An example search term used:

(“cerebral palsy” OR “physical disability” OR “physical impair*” OR “motor impair*” OR “muscular dystrophy” OR “muscular atrophy” OR “motor neuron disease” OR “stroke” OR “motor disorder” OR “brain injury” OR “parapleg*” OR “hemipleg*” OR “quadripleg*” OR “tetrapleg*” OR “dyspraxia” OR “Developmental Coordination Disorder” OR “spina bifida” OR “spinal cord injury” OR “gross motor” OR “Rett syndrome” OR “Angelman syndrome” OR “hydroceph*” OR “encepha*” OR “periventricular leukomalacia” OR “global development delay” OR “brain lesion” OR “preterm”)
AND (“infant” OR “infants” OR “baby” OR “babies” OR “newborn” OR “pediatric” OR “paediatric” OR “toddler”)
AND (“eye gaze technology” OR “eye tracking technology” OR “gaze based” OR “eye track*” OR “gaze track*” OR “eye control system” OR “eye gaze system” OR “gaze control technology”).

### 2.5. Study Screening

Search results were imported into Mendeley (Mendeley Ltd., 2020 London, UK, version 1.19.8) and Covidence (2026, Melbourne, VIC, Australia) for reference management and screening, respectively. Duplicate records identified by Covidence were removed prior to screening. During title and abstract screening, reviewers identified and removed an additional seven duplicate records that had not been detected automatically.

In Covidence, two reviewers (authors S.S. and P.K. or A.M.) independently screened titles, abstracts, and full texts against the eligibility criteria. Disagreements were resolved through discussion and, when necessary, consultation with a third reviewer. The percent agreement and Cohen’s kappa were reported for abstract screening and full text review.

### 2.6. Data Extraction

A data extraction and quality assessment template was created in Covidence and pilot tested by the authors. The finalized data extraction form was used to collect study characteristics (author, year, study design, and country), participant characteristics (e.g., age, sex, sample size, risk factors), intervention details (type of eye-tracking technology, duration, stimuli, and limitations), and outcomes (e.g., specific metrics reported like gaze behaviour, social attention which related to cognition, motor, social/emotional development). Where information was unclear or missing, this was recorded as “not reported”. Data extraction was performed independently by two reviewers using a standardized form in Covidence. Extracted data was compared for consensus. Discrepancies were resolved through discussion, with involvement of a third reviewer where required.

### 2.7. Risk of Bias Assessment

Two reviewers independently assessed the level of evidence and risk of bias using the Oxford Center for Evidence-Based Medicine (OCEBM) Level of Evidence [20] and Standard Quality Assessment Criteria for Evaluating Primary Research Papers [21]. For the OCEBM Level of Evidence, the ratings were as follows 1. Systematic reviews; 2. Randomized control studies; 3. Non-randomized control or follow up studies; 4. Case series or case–control studies; and 5. Mechanism-based reasoning. For the Standard Quality Assessment Criteria, the standard checklist was utilized. Discrepancies were resolved through discussion and consultation with a third reviewer when consensus could not be reached. Reporting bias was not formally assessed due to the limited number of studies and the substantial methodological heterogeneity across studies.

### 2.8. Data Analysis

The demographic data extracted from the papers was converted to standard units and the mean (standard deviation) was reported unless otherwise stated.

Due to heterogeneity in study populations, eye-tracking technology protocols, outcome measures, and study designs, a meta-analysis was not considered appropriate. Findings were therefore synthesized narratively in accordance with the review objectives. Extracted data were first organized according to the three review questions: (1) eye-tracking protocols and procedures used; (2) eye-tracking metrics reported; and (3) feasibility of eye-tracking technology. Study characteristics, participant demographics, and technology characteristics were summarized descriptively.

For the synthesis of eye-tracking protocols and procedures, information relating to hardware, testing environments, stimuli, session duration, frequency of use, and assessment procedures was extracted and summarized. Eye-tracking metrics were categorized according to the primary measure reported within each study, including gaze duration and gaze patterns, fixation-based measures, saccadic measures, visual assessment measures, and assistive technology performance measures. Feasibility outcomes were synthesized descriptively and included measures such as calibration success, accuracy, reliability, validity, user satisfaction, attention, fatigue, restlessness, and technology use over time.

Studies could contribute to multiple categories where more than one eye-tracking metric or feasibility outcome was reported. Common methodological limitations, sources of heterogeneity, and evidence gaps were also extracted and summarized narratively to identify priorities for future research.

## 3. Results

### 3.1. Study Characteristics

**Figure 1:**
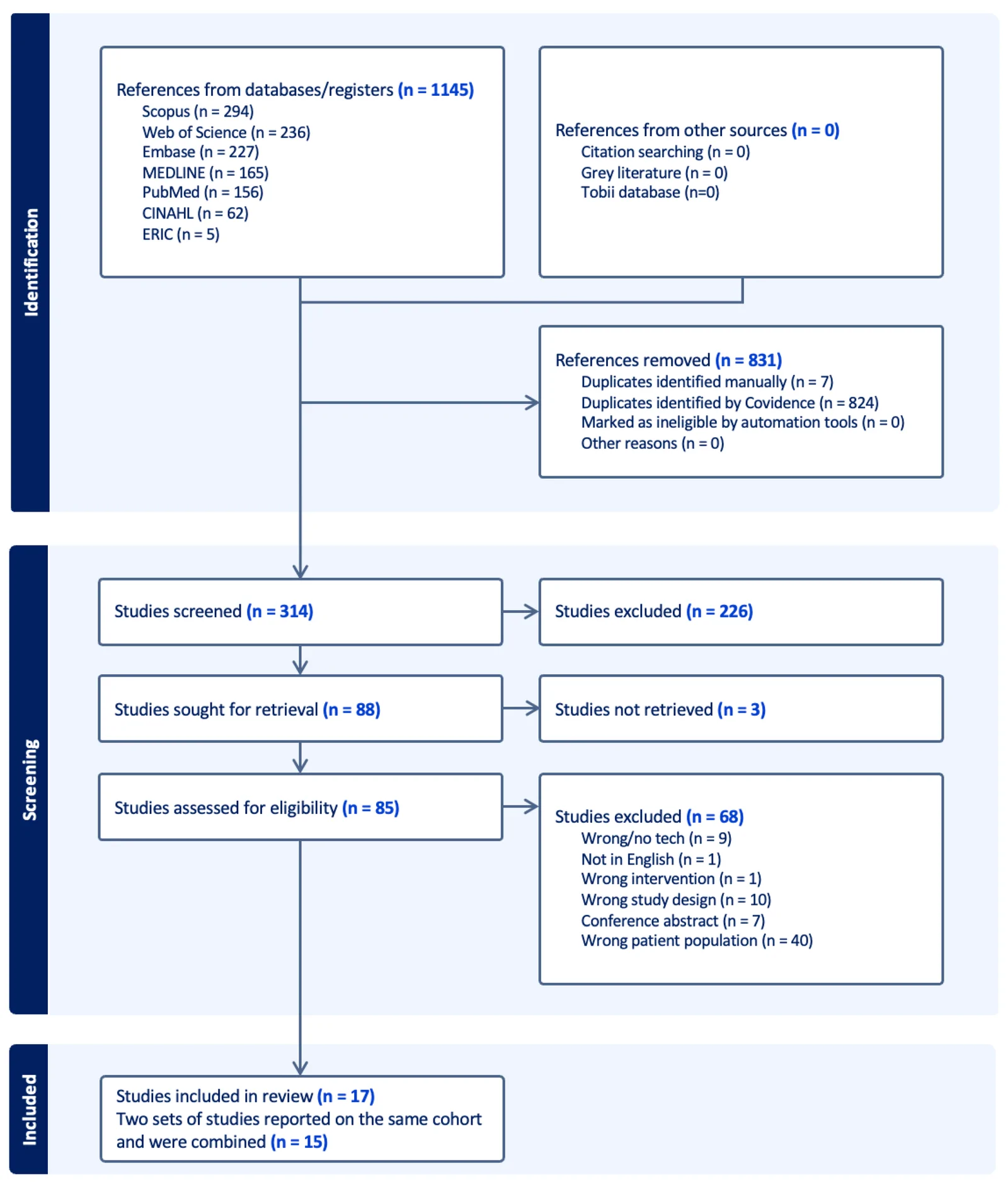
PRISMA flow chart of the systematic review study selection.

Initially, 1145 studies were identified using eight sources with 831 duplicates (Figure 1). The remaining 314 studies underwent title and abstract screening, with 226 studies excluded. Of the 88 full text studies screened, 3 could not be retrieved, and 68 were excluded. The main reasons for exclusion were the wrong population (*n* = 40), wrong study design (*n* = 10), no technology used (*n* = 9), and conference abstracts (*n* = 7). Seventeen studies were included in the final review. However, two sets of papers followed the same infant cohorts and were subsequently merged into two complete studies resulting in a final 15 studies (Refs. [10,22] and Refs. [23,24]). The inter-reliability was assessed with a percent agreement of 83.6% and Cohen’s Kappa of 0.56 for abstract screening and a percent agreement of 93.9% and Cohen’s Kappa of 0.82 for full text review.

The study characteristics are listed in detail in Table 1. The distribution of studies indicates that Sweden, the United States, Finland, and the Netherlands were the primary locations of investigation, with three studies in each country. Studies were also conducted in Estonia, Brazil, and the United Kingdom. All studies were published from 2016 onwards with a slight peak in 2019 and 2020.

This review included 667 infants with 51.1% (*n* = 341) boys, 45.9% (*n* = 306) girls, and one study did not report the gender of the 20 infants included [36]. The mean infant age from all studies was 10.8 ± 4.8 months, ranging from 4 months to 24 months. The sample sizes ranged from one to 123. The most common reason for presenting or being at risk for a motor disability was very preterm with complications (*n* = 5 studies), brain injury identified with imaging (*n* = 4 studies), cerebral palsy (*n* = 2 studies), epilepsy with suboptimal HINE score (*n* = 1 study), or cervical spinal cord injury (*n* = 3 studies). The extent of motor developmental delay due to cerebral palsy was assessed as severe in two studies (using Gross Motor Function Classification System Expanded & Revised (GMFCS E& IV-V) [22,26] moderate in one study [28], and mild in one study [32]. Three studies only reported the extent of brain injury rather than motor delay [30,36,37]. The remaining six studies did not assess the extent of motor disability or delay.

Most studies were cross-sectional studies (*n* = 4 studies), followed by cohort (*n* = 4), case series (*n* = 2), randomized controlled trial (*n* = 1), longitudinal (*n* = 1), correlational (*n* = 2), and quasi-experimental (*n* = 1). The studies were a range of quality based on OCEBM [20] and Standard Quality Assessment Criteria for Evaluating Primary Research Papers [21]. For the OCEBM Level of Evidence, one study was a randomized controlled trial at level two [28], ten were level three, and four were level four (Table 2). The standard quality assessment varied as well with 11 papers rated above 16/22 and 4 below 16/22. Papers rated below 16/22 had an unclear study design, did not control confounding, low sample size, unclear method of sample selection, and limited/no estimate of variance. Funding was reported in 12 out of 15 studies with no apparent association between funding source and study outcome.

### 3.2. Eye-Tracking Technology Protocols and Procedures

Twelve out of 15 studies used a Tobii eye tracker with versions including Tobi C12, X2-60, CX120, 60 XL/CL, X60, and Pro-X3-120. The remaining three studies used a self-developed eye tracker [28,30] or a Mirametrix S2 eye tracker [33] (Table 2). Despite the predominance of Tobii systems, studies varied substantially in equipment specifications, intervention, stimuli, study duration, and metrics reported.

Of the 15 studies included, eight were conducted at a hospital, three at home and in a clinic/lab, one study was at home/school, one study in a lab, and two studies did not specify the setting [32,33]. The diversity of study settings demonstrates its usability across wide settings; however, the lack of standardization limits comparison between studies.

Stimuli were presented for a range of 4.23 to 60 min with seven studies not reporting the exact duration. The stimuli presented were inconsistent among all studies ranging from toys [28], unspecified images [34], words [35], faces [25,29,30,33,37] or a mix of cartoons and geometric shapes [27,32,36]. Two studies also used Compass software 2.0 which evaluates keyboard/mouse/switch skills for access method selection [10,24]. The substantial diversity in stimulus selection reflected different study objectives and developmental domains assessed, preventing identification of a standardized stimulus protocol.

The duration and frequency of eye-tracking technology assessments varied considerably. Sessions were an average of 18.9 min ± 18.8 min. The overall duration of eye-tracking use ranged from a single assessment session lasting four minutes [27] to repeated daily use over extended periods [10,24,26]. Several studies (*n* = 7) did not specify the duration of eye-tracking technology usage and testing.

Overall, no standardized eye-tracking technology protocol was identified across studies. While Tobii systems were predominantly used, substantial heterogeneity existed in equipment, testing environments, stimulus selection, session duration, frequency of use, and reporting practices.

### 3.3. Eye-Tracking Technology Metrics Reported

The most common measures reported were gaze duration (*n* = 7 studies) an gaze patterns (*n* = 7) (Table 3). Studies also reported fixation frequency [27,35,37], visual assessments [24,31,32,36], and saccade latencies [27,29]. These metrics were often conducted in synergy with behavioural assessments to provide a more comprehensive understanding of the infant’s developmental trajectory. These metrics were used to assess developmental outcomes within cognition (*n* = 12 studies), social (*n* = 6), linguistic (*n* = 6), and motor development (*n* = 2).

#### 3.3.1. Gaze Duration and Patterns

Gaze duration and patterns were the most frequently reported metric across studies and used to infer cognitive, social, and language development [22,23,24,25,26,27,28,29,30,31,32,33,34,35,36,37]. In preterm infants, the proportion of time spent fixating on target stimuli was significantly lower at 12 months compared to full-term controls (34.7% vs. 44.1%, *p* < 0.001) [33]. Gaze duration and preferential looking were commonly used to assess social attention, with preterm infants at 7 months corrected age demonstrating a greater tendency to remain engaged with facial stimuli (mean = 0.55) than non-face patterns (mean = 0.24) [25]. Similarly, high-risk NICU infants demonstrated a preference for upright faces (47.4%) over inverted faces (34.3%), although this preference was reduced in infants with evidence of central nervous system injury [30].

Gaze metrics were also used to investigate language development. Exposure to parental speech in the NICU was positively associated with face preference and visual attention to faces, suggesting a potential marker of early social and language development [25]. Likewise, receptive lexical development at 15 and 18 months was positively associated with higher numbers of conversational turns and child vocalizations, whereas exposure to overheard adult speech was negatively associated with lexical processing accuracy [34]. In addition, gaze location and preferential looking towards matching scene–face pairs were used to assess relational memory and visual attention in infants with hypoxic-ischemic injury [37].

In studies investigating eye-tracking technology as an assistive device, gaze duration and performance measures demonstrated improvement over time. The median time-on-task decreased from 8 s at 9 months to 2 s at 20 months, indicating increased efficiency in gaze-based interactions [22,24,26].

#### 3.3.2. Fixation Measures

Fixation-based metrics included fixation frequency, fixation reliability, fixation duration, and fixation area [27,29,35,37]. These measures were used to assess visual attention, language development, social information processing, and developmental outcomes. In infants with early-onset epilepsy, those with typical developmental outcomes at two years demonstrated significantly higher fixation reliability (median = 0.96) than infants who later developed developmental delays (median = 0.58, *p* = 0.007) [29]. Higher fixation reliability and gaze shift probability were also associated with stronger language outcomes measured using the BSID-III and GMDS-III [29].

In preterm infants, fixation measures provided insights into visual attention and developmental variability. The median spontaneous fixation duration on cartoon stimuli was 1304 ms at one-year corrected age, although 15% of infants demonstrated abnormal fixation durations relative to normative references [30]. The median gaze fixation area for highly salient cartoon stimuli was 1.98 degrees [30]. Sex-related differences were also observed, with females demonstrating increasing attention towards the mouth region of upright faces across the first year of life, whereas males did not.

#### 3.3.3. Saccadic Measures and Visual Processing

Saccadic reaction time, gaze shift probability, fixation reaction time, and visual orienting measures were commonly used to investigate visual processing and the early detection of developmental delays [27,29,31,32,33,36]. Across studies, preterm infants demonstrated slower visual processing speeds, delayed reaction times, and reduced attentional shifting compared with full-term infants [27,31].

At 12 months, preterm infants exhibited significantly slower saccadic responses to target stimuli at a 550 ms interval than full-term controls (0.38 s vs. 0.30 s, *p* = 0.009) [31]. Similarly, reaction times to fixation were prolonged for intermediate-salience stimuli, including Cartoon (261 ms), Motion (686 ms, *p* = 0.025), and Form (1043 ms, *p* = 0.003) stimuli [31]. Infants with typical developmental outcomes following epilepsy also demonstrated higher gaze shift probabilities (median = 1.0) than infants who subsequently developed developmental delays (median = 0.70) [29]. Median reaction time variability for motion stimuli was reported as 97 ms at one-year corrected age [30].

Four studies reported that delayed visual processing measures were associated with brain injury and developmental delay in preterm infants [27,31,32,36]. Reduced stimulus detection and prolonged reaction times were particularly evident among infants with severe neonatal complications, including infant respiratory distress syndrome and intraventricular hemorrhage [31,32].

#### 3.3.4. Visual Assessment Measures

Visual assessment measures were reported in four studies and were primarily used to evaluate visual orienting functions, stimulus detection, and visual processing performance [24,31,32,36]. Eye-tracking assessments identified visual processing abnormalities that were not consistently detected using conventional visual assessment approaches. At 12 months corrected age, 38% of very preterm infants were identified as being at risk of visual processing dysfunction using eye-tracking measures, despite relatively few abnormalities being detected through conventional visual diagnostics [32]. Parents of infants identified as being at risk for visual processing dysfunction also reported significantly greater difficulties in mobility-related activities (*p* = 0.043) [32]. Reduced stimulus detection rates and prolonged reaction times were consistently reported among infants with significant perinatal risk factors, including respiratory failure and brain injury [31,32].

### 3.4. Early Detection and Assessment of Developmental Delays Using Eye-Tracking Technology

Four studies reported that delayed visual processing, measured using eye-tracking technology, was indicative of brain injury and developmental delay in preterm infants [27,29,31,32,33,36]. At 12 months corrected age, preterm infants demonstrated slower visual processing speed and reduced ability to shift attention compared to full-term infants, even after accounting for age-related effects [27]. Processing speed and proportion of target fixation were strongly associated with age, highlighting the sensitivity of eye-tracking metrics to developmental change. Similarly, van Gils et al. [36] found that at one year of age, slower fixation reaction times and minimum reaction times to moving stimuli were associated with structural brain damage, although these associations were not observed at two years of age. Across studies, eye-tracking was used to quantify eye-movement response times and looking patterns to infer visual processing speed and function.

Compared to age-matched controls, preterm infants also exhibited lower detection rates and higher median reaction times across multiple visual stimulus categories, including Cartoon, Form, and Motion stimuli [31]. Delayed reaction times were observed in 19% of preterm infants for Cartoon stimuli, 21% for Form stimuli, and 23% for Motion stimuli. These visuospatial attention and processing deficits were more prevalent in infants with perinatal risk factors, such as respiratory failure and hemorrhages, and were predictive of later executive functioning outcomes [27,31]. The median fixation reliability, gaze shift probability, and saccadic reaction time were also lower in 6-month-old infants with epilepsy compared to typically developing infants even after adjusting for infant age [29].

Further evidence indicated that preterm infants showed suboptimal performance in visually guided eye movement responses, characterized by reduced stimulus detection and prolonged reaction times, particularly for high- and intermediate-salience stimuli [31,32,36]. These results were associated with severe neonatal conditions, including infant respiratory distress syndrome, intraventricular hemorrhage (IVH), and other indicators of brain injury [31,32]. At 12 months corrected age, eye-tracking technology identified 38% of very preterm infants at risk for visual processing disorders, whereas conventional visual diagnostics detected very few deficits [32]. Longitudinal findings further demonstrated an increase in abnormal visual orienting functions by two years of age, suggesting that a single early assessment may not capture emerging or persisting visual processing deficits.

### 3.5. Feasibility of Eye-Tracking Technology in Infants at Risk of a Motor Disability

Feasibility outcomes were inconsistently reported across studies, with few studies evaluating more than one aspect of feasibility. Some measures reported include calibration success, accuracy, reliability, validity, user satisfaction, fatigue, and technology use over time.

Quantitative feasibility outcomes were reported in a small number of studies. Kooiker et al. [32] reported a 94% calibration success rate, an attention score of 3.46 (0.6)/5, a fatigue score of 2.98 (0.89)/5, and a restlessness score of 3.10 (1.13)/5. Borgestig et al. [22] reported good test–retest stability, consistency, and validity of eye-tracking technology used as an access method. Accuracy improved from approximately 80% to 100% over time, parental satisfaction was high (3.9; range 3.4–4.2 out of 5), and children developed a broader range of computer-based activities [22]. Similarly, Borgestig et al. [26] reported increased eye-tracking technology use and improved accuracy from approximately 80% to 100% between 9 and 26 months of age. The authors also reported an accuracy of 0.8 degrees for the Tobii eye tracker, which was comparable to the 0.5 degrees reported by Wagner et al. [37]. Eye-tracking technology was successfully used in infants as young as four months of age across a range of developmental and assistive technology applications [30].

Barriers to feasibility assessment were reported. Five studies included sample sizes below 20 [22,24,26,27,28]. The small sample sizes along with convenience sampling and minimal sample diversity limited feasibility and outcome comparison [24,25,26,27,28,29,30,31,32,33,34,35,36,37]. Technical issues including calibration difficulties, motion artefacts, and equipment-related errors were reported across multiple studies [25,29,30,32,36]. Infant behaviour also influenced data collection, with studies reporting difficulties related to inattention, fatigue, restlessness, and infant demeanour [24,25,26,27,28,29,30,31,32,33,34,35,36,37]. Kittler et al. [30] reported that 33.8% of trials were excluded because infants failed to attend to the screen for at least 60% of the testing period. Additional challenges included participant dropout [30,32,36], loss to follow-up [25,35,37], insufficient data collection [27], and inconsistent reporting across all studies. Sex, age, and stimuli motion impacted outcomes and findings [29,30]. Moreover, only five studies had a control group [27,29,31,32,37].

Overall, eye-tracking technology was generally feasible in infants with or at risk of motor disability, including infants as young as four months of age. However, feasibility reporting was inconsistent, and challenges relating to calibration, infant attention, movement artefacts, and incomplete reporting were commonly observed.

## 4. Discussion

This review examined the feasibility, metrics, and protocols associated with the use of eye-tracking technology in infants at risk of or with motor disabilities. These findings support a broader body of developmental science demonstrating that gaze behaviour provides may infer early neurocognitive processes [38,39].

### 4.1. Protocols, Procedures, and Methodological Considerations

A key finding of this review was the absence of standardized eye-tracking protocols for infants with or at risk of motor disability. Although 12 of the 15 studies used Tobii eye-tracking systems, substantial variability existed in hardware versions, testing environments, stimuli, session duration, and outcome measures. Stimuli ranged from faces and words to toys, cartoons, geometric shapes, and access-method software. Session durations ranged from approximately 4 to 60 min, with seven studies not reporting testing duration [27,28].

Sex-related differences were only examined in a small number of studies. One study reported increasing attention to the mouth region among females but not males during the first year of life [30], while males were disproportionately represented among untestable participants [28,30]. These findings suggest that biological sex may influence visual attention, task engagement, and eye-tracking data quality. However, the small number of studies examining sex-specific effects and limited sample sizes prevent firm conclusions. Consequently, the evidence does not currently support a single optimal protocol, highlighting the need for standardized procedures and reporting frameworks akin to Gredebäck et al. [40] but tailored to infants with motor disability.

### 4.2. Metrics Captured Through Eye-Tracking Technology

This review identified five broad categories of eye-tracking metrics: (1) gaze duration and gaze patterns, (2) fixation-based measures, (3) saccadic measures, (4) visual assessment measures, and (5) assistive technology performance measures. Gaze duration and gaze patterns were the most frequently reported metrics and were used across cognitive, linguistic, and social applications [24,25,26,27,28,29,30,31,32,33,34,35,36,37]. Fixation-based and saccadic measures were commonly used to assess visual attention, processing speed, attention shifting, and developmental risk [27,29,35,37]. Visual assessment measures were primarily used to evaluate visual orienting functions and visual processing performance [28,31,36]. The three studies evaluating eye-tracking technology as an assistive technology reported accuracy, time-on-task, and access-method performance measures [22,24,26].

Across studies, these metrics were used to investigate cognitive, language, social, motor, and visual processing outcomes. Delayed reaction times, reduced attention shifting, and altered gaze behaviours were associated with developmental delay, brain injury, epilepsy, and prematurity [27,29,31,32,36]. Similarly, gaze duration, gaze patterns, and preferential looking measures were used to investigate language processing, social attention, and memory-related processes [25,30,34,35,37]. These findings support previous literature reporting that gaze behaviour changes with age and early neurodevelopmental processes [39,41].

A key finding of this review was the substantial heterogeneity in metric selection, definitions, and reporting. While gaze duration and gaze patterns were commonly reported, several metrics frequently used in the broader eye-tracking literature, such as pupil diameter, visual anticipation, and detailed fixation duration measures, were infrequently reported or absent [16]. Consequently, no core set of eye-tracking metrics could be identified for infants with or at risk of motor disability, highlighting the need for more standardized outcome selection and reporting.

### 4.3. Feasibility of Eye-Tracking Technology

The available evidence suggests that eye-tracking technology may be feasible both as an assessment tool and as an access method. However, evidence remains limited by small sample sizes, heterogeneous populations, and variable reporting of outcomes. As an assessment, it provides objective, high-resolution measures of early executive skills, visual attention, processing speed, fixation, and looking times [27,31]. In naturalistic contexts, such as play, eye-tracking technology captured first-person perspectives on attention strategies, hand activity, and object manipulation that are otherwise difficult to measure using traditional observational methods [28]. These capabilities align with prior work reporting that eye-tracking may detect early differences in infants with neurodevelopmental risk, when adjusted for confounders such as age and sex [27,31,41].

As an access method, studies report high user satisfaction and improved accuracy over time [22,24,26]. These longitudinal findings suggest that some visual processing abnormalities may emerge beyond a single assessment time point [32]. These findings echo broader assistive technology research showing that eye-tracking technology can enhance communication and autonomy when implemented with appropriate training and environmental support [23,42]. The few intervention studies reported increases in activity repertoire, technology use duration, and goal attainment following structured interventions with professional support [22,26].

No studies reported metrics that were unobtainable in infants at risk or with motor disability. However, some measures (e.g., visual anticipation, pupil diameter, fixation duration) were reported infrequently or not at all. This highlights the need for a wider range of metrics to confirm if eye-tracking is as feasible for infants with motor disabilities as it is for typically developing infants [43].

### 4.4. Limitations and Future Directions

Meta-analysis was not possible due to substantial heterogeneity in study design, participant populations, eye-tracking systems, stimuli, testing procedures, and outcome measures. Findings were therefore synthesized narratively according to the review objectives of protocols, metric reporting, and feasibility assessment. A formal certainty-of-evidence framework such as GRADE was not applied because the included studies reported heterogeneous outcomes and used diverse methodologies, preventing meaningful assessment of certainty across comparable outcome domains. The heterogeneity underscores the need for more rigorous, longitudinal, and inclusive research before definitive conclusions can be drawn. Common practical challenges include small samples [22,24,26], posture-related calibration difficulties in infants with motor problems [28], and infant non-compliance (distraction, sleepiness, crying), which leads to dropped trials; males were disproportionately represented among untestable cases [28,30]. The variability in equipment, stimuli, duration, and metrics made comparison challenging. There was also a socioeconomic skew toward high income settings. Future work should prioritize:

- Standardized calibration and testing protocols tailored to infants with motor disabilities;
- Longitudinal designs to track developmental trajectories;
- Culturally and socioeconomically diverse participants;
- Integration of multimodal data (e.g., motion capture, physiological measures);
- Evaluation of real-world implementation in clinical and educational settings.

Addressing these limitations would support future meta-analysis and support clinical implications, which was not possible in this systematic review.

### 4.5. Clinical Implications

The available evidence suggests that eye-tracking technology may provide an objective method for assessing visual attention, processing speed, gaze behaviour, and assistive technology performance in infants with or at risk of motor disability [22,25,28]. However, the current evidence base remains insufficient to support routine clinical implementation due to substantial methodological heterogeneity and the predominance of small observational studies. It has potential to detect subtle deficits in processing speed, attention, and memory [27,37]. These may be missed using conventional behavioural methods and may provide an indication of infant developmental progress.

## 5. Conclusions

Eye-tracking technology shows promise as an objective approach for investigating infant development through eye-tracking metrics, including in infants with or at risk of motor disability. Across the included studies, eye-tracking was found to be feasible in a range of infant populations and provided valuable insights into attention, gaze behaviour, perception, and early developmental processes. However, the current evidence base remains limited by the small number of studies, generally small sample sizes, and substantial heterogeneity in participant populations, eye-tracking technologies, protocols, and reported outcome measures. As a result, direct comparisons across studies remain challenging and conclusions regarding clinical utility should be interpreted with caution. Future research should prioritize larger, well-designed studies with standardized protocols, outcome measures, and reporting practices to improve comparability and strengthen the evidence base.

## Figures and Tables

**Table 1:** Study characteristics for included studies.

Reference	Country	Study Design	Study Setting	Motor Disability or Risk Factor	Age in Months (SD)	Sample Size (*n*)	Boys (*n*)	Girls (*n*)
[25]	Finland, Estonia	Quasi-experimental study	Hospital	IVH, PVL, preterm	8.25 (0.13)	63	39	24
[22]	Sweden	Case series	Home and school	Cerebral palsy with GMFCS IV & MACS IV	12	1	0	1
[26]	Sweden	Longitudinal study with a before and after design	Home or pediatric centre	Cerebral palsy & cervical spinal cord injury with GMFCS IV & MACS IV	10	1	0	1
[27]	UK	Cross sectional study	Hospital	Very preterm, IVL, PVL, BSID Cognitive score 100 (7.7)	12.9 (1.1)	16	9	7
[28]	United States	Randomized controlled trial	Home or lab	Identified motor delays, very preterm, IVH, PVL, GMFCS I (7); II (5), III (8)	15 (6.9)	20	Not reported	Not reported
[24]	Sweden	Case series	Home or clinic/rehab	Cerebral palsy & high spinal cord injury	9	1	1	0
[29]	Finland	Cohort study	Hospital	Sub optimal HINE score, BSID-III score <85, epilepsy	6.1 (range 4.6–7.7) 12.2 (range 12–12.6)	51	29	22
[30]	United States	Longitudinal study	Hospital	Three groups; (1) no abnormalities, (2), abnormalities in brain stem, (3) global abnormalities observed during a cranial ultrasound	9.9 (3.5)	108	48	60
[31]	The Netherlands	Cohort study	Not reported	Very preterm, IVH, PVL, ROP, respiratory distress syndrome, BPD, low birth weight, low Apgar scores, brain damage visible on MRI	12.93	123	63	60
[32]	The Netherlands	Cross sectional study	Hospital	IVH, cerebral palsy, meningitis, sepsis, brain anomalies. Brain damage global score 4.54(3.28) Bayley scale: Cognitive 105 (7.8) Cognitive classification 3.3 (0.5) motor score 99(15) motor classification 3(0.7)	6–18	48	30	18
[33]	Brazil	Cross sectional study	Not reported	IVH, low birth weight, ROP, BPD, sepsis, respiratory distress syndrome	8.0 (0.8)	31	12	19
[34]	Finland	Cohort study	Hospital	IVH, very preterm, low birth weight, multiples, BPD, ROP	8.5 (*n* = 43) 15 (*n* = 35) 18 (*n* = 35)	43	24	19
[35]	Finland	Other: correlational study	Hospital	Very preterm, IVH, ROP, BPD, multiples	12 (*n* = 22) 15 (*n* = 22) 18 (*n* = 23)	25	12	13
[36]	The Netherlands	Prospective cohort study	Hospital	IVH, PVL, very preterm, low birth weight, brain injury	12 (*n* = 82) 24 (*n* = 59)	112	58	54
[37]	United States	Cross sectional study	Lab	HII, Sarnat stage I–II	6.7(0.5) 12 infants 12.6(0.5) 12 infants	24	16	8

Abbreviations: BPD, bronchopulmonary dysplasia; ROP, retinopathy of prematurity; IVH, intraventricular hemorrhage; PVL, periventricular leukomalacia; HII, hypoxic-ischemic injury; very preterm is <32 weeks.

**Table 2:** Eye-tracking technology protocols and quality.

Reference	Eye-Tracking Technology Used	Stimuli Presented	Stimuli Duration	Technology Use Duration (min)	OCEBM Level of Evidence	Standard Quality Assessment
[25]	Tripod-mounted 30 Hz video camera behind a monitor (Canon Legria, HFR806) with an Eye Tribe; Copenhagen, Denmark or Tobii X2-60 camera	Faces or non-faces pattern	Four 12-trial blocks (48 trials total)	Not reported	3	20/22
[22]	Tobii C12 or P10	Compass software activities (games, communication, counting, internet search, music)	Not reported	Daily	4	14/22
[26]	Tobii C12	Compass calibration task selecting animal targets using mouse control	daily use (25–60 min/day); assessments at baseline, 5, 9–11, and 15–20 months	Not reported	4	16/22
[27]	Tobii X60 with E-prime software	Animated geometric target characters with grey diamond distractors	36 trials; prime and probe 2000 ms each; Inter stimulus interval 67–550 ms; 1500 ms inter-trial interval	4.23	3	17/22
[28]	Head-mounted unit with a scene camera and an eye camera affixed	Free play with toys	2 min each, 1 h total	60	2	18/22
[24]	C12 Tobi Technology	Compass games and communication software	25–38 min/day, 5 days/week	31.5	4	8/22
[29]	Tobi Pro-X3-120	Geometric shapes and still faces	Not reported	Not reported	3	21/22
[30]	TOBII X120 eye tracker and SONY Handycam CMOS camera for monitoring	Faces (static/moving; silent or with synchronized vocalization)	Total presentation time 80 s	Not reported	3	22/22
[31]	Tobi T60XL	Geometric shapes and high-salience cartoons	Cartoons shown 16 × 10 s; other stimuli 4 × 4 s	8	3	19/22
[32]	Tobii T60 XL or Tobii X3	Geometric shapes, high-salient cartoons and moderate-salient motion stimuli	4 s per stimuli	15	3	18/22
[33]	Mirametrix S2 Eye Tracker version 2.0.0.057	Faces and boards with social vs. non-social figures (direct/indirect gaze)	Six boards presented for 5 s each in fixed sequence	Not reported	4	16/22
[34]	Tobi X2-60	Images paired with matching spoken words	Not reported	Not reported	3	21/22
[35]	Tobi X2-60	Paired images (target vs. non-matching distractor)	Images shown ~2 s before audio and ~1 s after; 5 test blocks	10	3	21/22
[36]	Tobi T60XL	Geometric shapes and cartoons	Cartoon shown 16 times; motion targets shown 4 times per sequence	Not reported	3	20/22
[37]	Tobii T60 monitor	Faces and scenes in learning and relational memory tasks	Familiarization 25 s; test 20 s after 2 min delay; total ~3 min Relational Memory Task: 24 s learning trials, 12 test trials at 8 s/blockTotal of ~6.5 min	16	3	19/22

Abbreviations: GMFCS, Gross Motor Function Classification System; MACS, Manual Ability Classification System; CFCS, Communication Function Classification System; BSID, Bayley Scales of Infant and Toddler Development.

**Table 3:** Metrics reported using eye-tracking technology in infants with or at risk of a motor disability.

Eye-Tracking Technology Metric	No. of Studies	Developmental Domains Investigated	References
Gaze duration	7	Cognition, Linguistic, Motor, Social	[24,25,28,30,33,34,35]
Gaze patterns	7	Cognition, Linguistic, Social	[25,27,28,30,33,34,37]
Access method performance	3	Cognition, Linguistic, Social	[22,24,26]
Fixation frequency	3	Cognition, Linguistic	[27,35,37]
Gaze fixation	1	Cognition, Linguistic	[29]
Gaze shifts	1	Cognition, Linguistic	[29]
Saccadic measures (reaction time/latency)	2	Cognition, Linguistic	[27,29]
Visual acuity/visual assessment	4	Cognition, Motor	[24,31,32,36]

## Data Availability

The original contributions presented in this study are included in the article. Further inquiries can be directed to the corresponding author.

## Supplementary Materials

The following supporting information can be downloaded at: https://www.mdpi.com/article/10.3390/children13081011/s1, File S1: PRISMA checklist [44].

## Abbreviations

The following abbreviations are used in this manuscript:

BSID-III	Bayley Scales of Infant and Toddler Development, Third Edition
DAYC-2	Developmental Assessment of Young Children, Second Edition
AIMS	Alberta Infant Motor Scale
PICO	Population–Intervention–Comparison–Outcome
PRISMA	Preferred Reporting Items for Systematic Reviews and Meta-Analyses
HINE	Hammersmith Infant Neurological Examination
OCEBM	Oxford Centre for Evidence-Based Medicine
GMDS-III	Griffiths Mental Development Scales, Third Edition (Griffiths Scales)
IVH	Intraventricular Hemorrhage
